# Adherence to the Planetary Health Diet Index and Obesity Indicators in the Brazilian Longitudinal Study of Adult Health (ELSA-Brasil)

**DOI:** 10.3390/nu13113691

**Published:** 2021-10-20

**Authors:** Leandro Teixeira Cacau, Isabela Martins Benseñor, Alessandra Carvalho Goulart, Leticia Oliveira Cardoso, Paulo Andrade Lotufo, Luis A. Moreno, Dirce Maria Marchioni

**Affiliations:** 1 Department of Nutrition, School of Public Health, University of São Paulo, São Paulo 01246-904, Brazil; lcacau@usp.br (L.T.C.); marchioni@usp.br (D.M.M.); 2 Growth, Exercise, Nutrition and Development (GENUD) Research Group, Faculty of Health Sciences, University of Zaragoza, 50009 Zaragoza, Spain; 3 Clinical and Epidemiological Research Center, University Hospital, University of São Paulo, São Paulo 05508-000, Brazil; isabensenor@gmail.com (I.M.B.); agoulart@hu.usp.br (A.C.G.); palotufo@usp.br (P.A.L.); 4 Department of Clinical Medicine, Faculty of Medicine, University of São Paulo, São Paulo 01246-903, Brazil; 5 National School of Public Health, Fundação Oswaldo Cruz, Rio de Janeiro 21041-210, Brazil; leticiaocar@ensp.fiocruz.br; 6 Centro de Investigación Biomédica en Red de Fisiopatología de la Obesidad y Nutrición (CIBEROBN), Instituto de Salud Carlos III, 28040 Madrid, Spain; 7 Instituto Agroalimentario de Aragon (IA2), 50013 Zaragoza, Spain; 8 Instituto de Investigación Sanitaria de Aragón, 50009 Zaragoza, Spain

**Keywords:** EAT-Lancet diet, sustainable diet, diet quality, obesity

## Abstract

The EAT-Lancet Commission has proposed a model diet to improve the health of human beings and that of the planet. Recently, we proposed the Planetary Health Diet Index (PHDI) to assess adherence of the population to this model diet. In this study, we aimed to evaluate adherence to the PHDI and obesity outcomes using baseline data from 14,515 participants in the Brazilian Longitudinal Study of Adult Health (ELSA-Brasil). The dietary data were assessed using a 114-item FFQ. Body mass index (BMI) and waist circumference (WC) were both used continuously and categorized. Linear and multinomial regression models adjusted for potential confounding factors were performed to assess the relationship between adherence to PHDI and outcomes. An inverse association was observed between adherence to PHDI and obesity indicators. Individuals with high adherence to the PHDI had lower BMI (β−0.50 95% CI−0.73:−0.27) and WC (β−1.70 95% CI−2.28:−1.12) values. They were also 24% less likely to be overweight (OR 0.76 95% CI 0.67:0.85) or obese (OR 0.76 95% CI 0.65:0.88), and they were 14% and 27% less likely to have increased WC (OR 0.86 95% CI 0.75:0.98) or substantially increased WC (OR 0.73 95% CI 0.64:0.83) than those with lower adherence. Our results showed that higher adherence to the PHDI may decrease obesity indicators.

## 1. Introduction

Early in 2019, the EAT-Lancet Commission on “Healthy Diets from Sustainable Food Systems” (EAT-Lancet) published a scientific report on sustainable diets and proposed a healthy reference diet for human and planetary health, called the "planetary health diet" (PHD) [1]. The recommendations of this reference diet are based on human health impacts and the environmental impacts related to the food system. Briefly, this diet is focused on a predominant consumption of vegetables, fruits, whole grains, legumes, nuts, and unsaturated oils, includes a low-to-moderate consumption of seafood and poultry, and includes no or a low consumption of red meat, processed meat, added sugar, refined grains, and starchy vegetables [1].

The EAT-Lancet report estimates that following the recommendations for a healthy and sustainable diet could prevent 11 million deaths per year [1]. The proposed reference diet has been debated in scientific research [2] and it has been studied as a model for healthy and sustainable food intake in the context of local food cultures [3,4]. In addition, some authors have also compared the EAT-Lancet diet with the recommendations for a healthy diet in the US [5] and with the food consumption of the Indian population [6]. In terms of cost, the EAT-Lancet diet is affordable, except for the inhabitants of low–middle income countries, where it would cost around 89% of household income per capita [7,8]. 

The main purpose of the EAT-Lancet diet is to improve the health of the population and the planet, and for that, the report confirms that this reference diet is nutritionally balanced and has a low environmental impact. However, one study noted that adopting the PHD on a global scale would have different impacts on greenhouse gas emissions (GHGE) [9]. Studies that assess adherence to the EAT-Lancet recommendations in different scenarios and countries are of interest, and some initiatives to assess adherence to the EAT-Lancet recommendations have been proposed. The first was the EAT-Lancet diet score, which considers 14 food groups in a binary score and was inversely associated with ischemia heart disease and diabetes [10]. However, this index uses a binary score, which does not allow an accurate assessment of the adherence to the EAT-Lancet recommendations; in addition, it does not include all intermediate values and interchangeable groups, as proposed in the EAT-Lancet report itself [11]. 

Consequently, we recently proposed the Planetary Health Diet Index (PHDI), which consists of 16 components that score proportionally and consider all EAT-Lancet food groups in addition caloric density [12]. PHDI scores were associated with higher overall dietary quality and lower GHGE emission, in addition to differences according to sex, age, smoking status, and physical activity. [12]. However, the PHDI has not yet been tested in terms of associations with health outcomes. Thus, in this study, we aimed to assess the relationship between adherence to the EAT-Lancet recommendations assessed according to PHDI and obesity outcomes, as it is one of the main public health issues worldwide and an established associated factor in the development of non-communicable chronic diseases, such as diabetes and cardiovascular disease [13,14,15]. To achieve this purpose, we used data from the Brazilian Longitudinal Study of Adult Health (ELSA-Brasil), a well-established ongoing cohort study in Brazil.

## 2. Materials and Methods

### 2.1. Study Design

**Figure 1:**
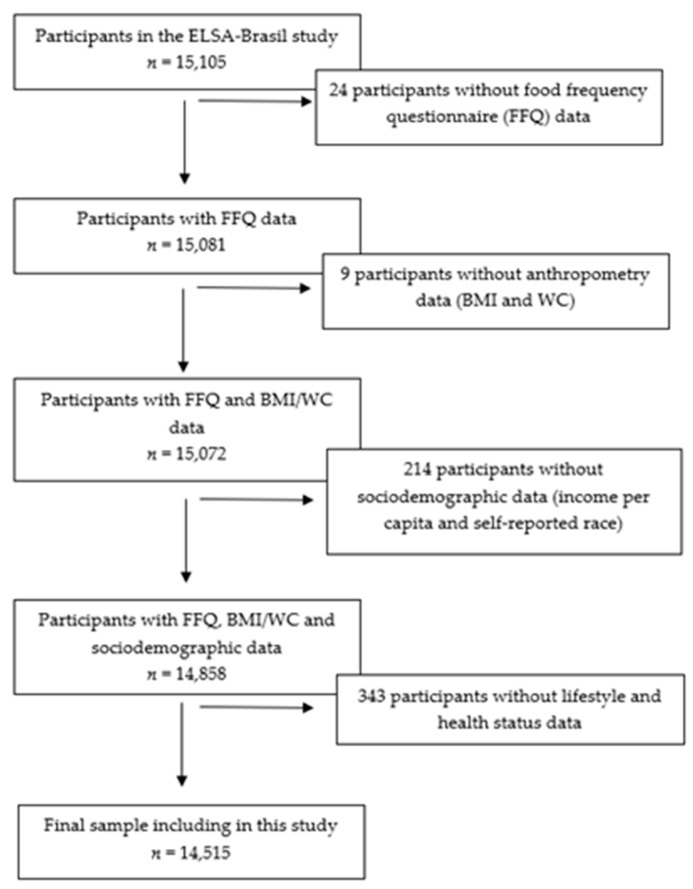
Flow chart of the sample included in the study process. ELSA-Brasil, 2008–2010.

This study is a cross-sectional analysis of the baseline data from ELSA-Brasil, a multicenter and ongoing cohort study conducted in six Brazilian cities (São Paulo, Rio de Janeiro, Salvador, Porto Alegre, Belo Horizonte and Vitoria) from three major Brazilian regions (Northeast, Southeast, and South). The study design and data collection were described previously [16,17,18,19]. Briefly, ELSA-Brasil enrolled 15,105 civil servants from five universities and one research institute, with the following exclusion criteria: current or recent (4 months prior to the first interview) pregnancy, intention to quit working at the institution in the near future, severe cognitive or communication impairments, and, if retired, residence outside of a study center. Baseline data from ELSA-Brasil were collected between August 2008 and December 2010. ELSA-Brasil was approved by the research ethics committees of all the research centers. All the participants volunteered and signed an informed consent form. For the present analysis, we disregarded participants without food consumption, anthropometric, or covariates data; thus, the final sample for analysis included 14,515 individuals (Figure 1).

### 2.2. Diet Assessment

Food consumption was assessed using a previously developed and validated semi-quantitative FFQ with 114 food items [20,21]. This FFQ comprised the past 12 months and the questions were structured into three sections: (1) food products/food preparation; (2) measures of consumed products; and (3) consumption frequencies with eight response options (more than 3 times a day, 2–3 times a day, once a day, 5–6 times a week, 2–4 times a week, once a week, 1–3 times a month, and never/almost never). 

The daily consumption of each FFQ item (in g/day) was obtained by multiplying the portion size by the corresponding frequency. The food measurements were then converted into nutrient intake using the United States Department of Agriculture (USDA) Food Composition Database, except when its values were outside the range of 80% to 120% of those described in the Brazilian Table of Food Composition, where the latter reference was used. 

### 2.3. Planetary Health Diet Index

The Planetary Health Diet Index (PHDI) is based on the recommendations of the reference diet proposed by the EAT-Lancet Commission [1]. Its development and validation process were described previously [12]. Briefly, PHDI is a calorie-based index, since the ranges and midpoints proposed for each food group in the EAT-Lancet report are calculated as their energetic contribution to the reference diet, which makes it possible to assess adherence to EAT-Lancet recommendations regardless of the amount of calories consumed [12]. The index has 16 components, divided into 4 categories: (1) adequacy components (nuts and peanuts, legumes, fruits, total vegetables, and whole cereals); (2) optimum components (eggs, dairy products, fish and seafood, tubers and potatoes and vegetable oils); (3) ratio components (dark green vegetables/total vegetables and red and orange vegetables/total vegetables) and (4) moderation components (red meat, chickens and substitutes, animal fats and added sugars). 

**Figure 2:**
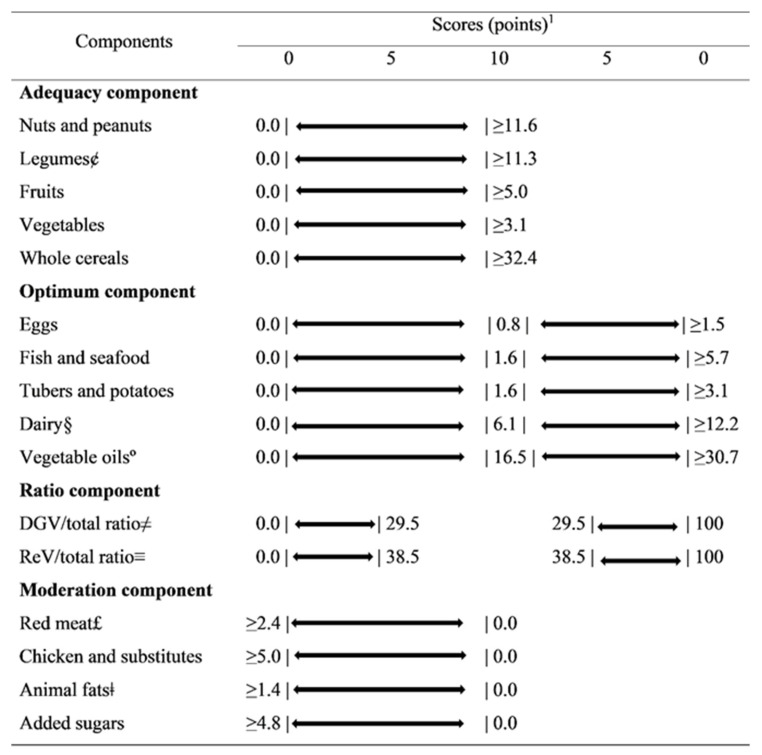
Planetary Health Diet Index components, standards for scoring (caloric densities) and corresponding points values. ^1^ All values expressed as caloric densities from the reference diet proposed by the EAT-Lancet Commission. The bars represent the limits. £ Red meat: beef, lamb and pork. ¢ Legumes: beans and soy. § Dairy: excluding dairy fats. ° Unsaturated oils: including palm oil. ≠ DGV/total ratio: ratio between the energy intake of dark green vegetables (numerator) and the total of vegetables (denominator) multiplied by 10. ≡ ReV/total ratio: ratio between the energy intake of red and orange vegetables (numerator) and the total of vegetables (denominator) multiplied by 10. ǂ Animal fat: lard, tallow and dairy fats. DGV/total ratio: dark green vegetable/total ratio. ReV/total ratio: red vegetable/total ratio.

Each one of the 16 components can score proportionally between 0 and 5 or 10 points. The components in the adequacy, optimum and moderation categories can reach a maximum of 10 points, while the ratio components can score a maximum of 5 points, resulting in a total score from 0 to 150 points. More information regarding the cutoff points and scoring criteria can be obtained from Cacau et al. [12]. Figure 2 presents the PHDI components, cut-off points and scoring criteria. Table S1 shows examples of the foods and ingredients included in the PHDI components. 

### 2.4. Outcome’s Assessment

The anthropometric measures of weight, height, and waist circumference (WC) were obtained using international criteria and standards techniques [18]. The body weight was measured with the subject barefoot, fasted, and wearing a standard uniform over their underwear. An electronic scale (Toledo^®^, model 2096PP) was used, with a capacity of 200 kg and a precision of 50 g. The height was measured with a wall stadiometer (Seca^®^, Hamburg, BRD) with a precision of 1 mm, attached to the wall, with the individual in a supine position, barefoot, leaning their head, buttocks, and heels against the wall and staring in the horizontal plane. The WC was measured with the participant fasted and with an empty bladder, standing upright, breathing normally, with their feet together, part of their standard uniform lifted, and with their arms crossed in front of their chest. The measurement was taken at the midpoint between the crest and the lower edge of the costal arch, using an inextensible tape. 

The Body Mass Index (BMI) was calculated as weight (kg) divided by squared height (m^2^) [18]. The BMI and WC were treated as continuous variables and used to generate measures of obesity and abdominal obesity, respectively. BMI values <25 kg/m2 were considered adequate, ≥25 kg/m^2^ to <30 kg/m^2^ was considered overweight, and ≥30 kg/m^2^ as obesity [22]. A WC <80 cm for women and <94 cm for men was classified as adequate, ≥80 cm to <88 cm for women and ≥94 cm to 102 cm for men as increased WC, and ≥88 cm for women and ≥102 cm for men as an indicator of substantially increased risk [22].

### 2.5. Covariates

Each participant was interviewed at his or her workplace and visited the research center for clinical examinations, according to standard protocols. These interviews focused on sociodemographic characteristics, which were obtained using a general questionnaire [16]. The sociodemographic characteristics used were: sex, age, self-reported race and income per capita. The participants were classified according to sex (men and women) and according to age, as adults (34–59 years) and elderly (≥60 years). Self-reported race was classified as white, brown, black or other (Asian and Indigenous), according to previous ELSA-Brasil studies [17,18]. The self-reported income per capita was calculated as the total family monthly income divided by the number of family members, and the result then divided into terciles.

Smoking was stratified by nonsmokers, ex-smokers, and current smokers. Alcohol consumption was dichotomized into yes or no based on excessive alcohol consumption (those with an ethanol consumption ≥210 and ≥140 g/week, for men and women, respectively) [17,23]. The levels of physical activity during leisure time were classified as low, moderate, or vigorous, according to the International Physical Activity Questionnaire (≥150 min/week of moderate activity or ≥75 min/week of vigorous activity) [24]. 

Hypertension was defined as the reported use of medications to treat hypertension, a systolic blood pressure ≥140 mmHg, or a diastolic blood pressure ≥90 mmHg [19]. Diabetes was defined as a medical history of diabetes mellitus, the reported use of medications to treat diabetes mellitus, a fasting serum glucose >126 mg/dl, HbA1c levels 6.5%, or a 2 h oral glucose tolerance test >200 mg/dl [19]. Dyslipidemia was defined as the reported use of lipid-lowering treatment or an LDL cholesterol level >130 mg/dl [19].

### 2.6. Statistical Analysis

We compared the baseline characteristics of the participants according to quintiles of the PHDI scores. We calculated means and standard deviations (SD) or percentages for each variable across the quintiles. ANOVA or Pearson's chi-square tests were used to assess the statistical significance of differences between means or proportions, respectively.

Multiple linear regression models were used to assess the associations between PHDI scores and BMI and WC, respectively. Multiple multinomial logistic regression models were used to determine associations between the PHDI scores and the categories of BMI and WC. We used the PHDI scores, both categorized into quintiles and continuous (increase of 10 points in the total PHDI score), as explanatory variables. All the models were presented as age-adjusted models and fully adjusted models with sex, self-reported race, per capita family income, smoking, sporadic alcohol intake, diabetes, hypertension, dyslipidemia, total energy intake and dietary changes in the last six months, in addition to the age adjustment. We performed a sensitivity analysis excluding individuals below p1 and above p99 for total energy intake, as under- or over-reporters, respectively.

All the statistical analyses were performed using STATA^®^ (Statistical Software for Professionals, College Station, TX, USA), version 14.2 and the *p*-value <0.05 was considered statistically significant.

## 3. Results

The baseline characteristics of the participants, according to the quintiles of the PHDI scores, are summarized in Table 1. Those with a higher PHDI (fifth quintile, Q5) were more likely to be elderly, white, or of other race, non-smokers, with a higher per capita income, no excessive alcohol consumption, higher physical activity levels, diabetes, hypertension, and lower total energy intake. The PHDI quintiles were also directly associated with lower BMI and WC values and lower prevalence of obesity and abdominal obesity. 

Table 2 presents crude and adjusted analyses of the continuous association between the quintiles of PHDI scores and BMI and WC. According to the fully adjusted analyses, the subjects with the higher PHDI scores (5th quintile, 5Q) demonstrated values of −0.50 kg/m^2^ for BMI (95%CI−0.73:−0.27) and −1.70 cm (95% CI−2.28:−1.12) for WC, when compared to individuals in the first quintile. An overall significant linear trend was observed for the association between the quintiles of the PHDI scores and the two outcomes (*p* < 0.001), even with the non-significance of some terciles. In addition, there was a decrease of −0.15 kg/m^2^ (95% CI−0.21:−0.08) in the BMI and −0.52 cm (95% CI−0.68:−0.36) in the WC for each 10-point increase in the PHDI score in the fully adjusted models.

The multinomial regression models suggested that higher PHDI scores (5th quintile, 5Q) were associated with a lower likelihood of obesity when compared with those in the first quintile (Table 3). The subjects with the higher PHDI scores were 24% less likely to be overweight (95% CI 0.67:0.85) or obese (95% CI 0.65:0.88) in the fully adjusted models. Similar results were found for abdominal obesity (Table 4), with those with higher PHDI scores being 14% and 27% likely to have increased abdominal obesity (95% CI 0.75:0.98) and substantially increased abdominal obesity (95%CI 0.64:0.83), respectively. In the fully adjusted model, a 10-point increase in PHDI score was associated with a 7% lower likelihood of being overweight (95% CI 0.90:0.97) and an 8% lower likelihood of obesity (95% CI 0.88:0.96) (Table 3), and with a 5% lower likelihood of having increased abdominal obesity (95% CI 0.92:0.99) and a 9% lower likelihood of having substantially increased abdominal obesity (95% CI 0.87:0.94) (Table 4). A significant linear trend was observed for the association between the quintiles of the PHDI scores and being overweight (*p* < 0.001), obesity (*p* < 0.001), and substantially increased abdominal obesity (*p* < 0.001). 

In the sensitivity analysis, excluding individuals below p1 and above p99 from total energy intake had little effect on the magnitude of the associations, keeping the same directions for the associations between obesity and PHDI (Tables S2–S4).

## 4. Discussion

This is the first study to assess the association of a dietary caloric density index that scores gradually to assess adherence to EAT-Lancet recommendations with obesity outcomes. As a result, we observed an inverse association between adherence to EAT-Lancet recommendations–evaluated by higher scores on the planetary health diet index (PHDI)–and obesity indicators assessed by BMI and WC, both continuously and categorized, in participants from a large multicentric ongoing cohort study in Brazil. In our previous study, with the same population, we observed that higher PHDI scores were associated with higher overall dietary quality and lower GHGE emissions [12]. These results suggested that the EAT-Lancet [1] recommendations for a healthy and sustainable diet can be beneficial both for the environment and for human health, as they are associated with a lower risk of being overweight or obese, both of which are important factors associated with other chronic diseases, such as diabetes and cardiovascular diseases. 

Few studies evaluated the association between healthy and sustainable diets and obesity and, as far as we know, only two studies evaluated the relationship between the EAT-Lancet recommendations and obesity indicators [10,25]. Shamah-Levy et al. [25] proposed an index based on the EAT-Lancet recommendations. Briefly, this index features 13 components that consider the percentage of energy from the EAT-Lancet cutoff points and uses an overall score from 0 to 13 points. In an analysis with a representative sample of 11,506 Mexican adults aged 20 to 59 years and of both genders, Shamah-Levy et al. [25] observed a lower prevalence of excess weight and obesity in individuals with ≥9 points in the index. After statistical adjustments, men with ≥9 points on the index were 14.3% less likely to be obese. 

In another study, which evaluated 46,069 participants enrolled throughout the UK, in the European Prospective Investigation into Cancer and Nutrition (EPIC)-Oxford study, Knuppel et al. [10] observed a decrease of 1.4 kg/m^2^ in the BMI of individuals with ≥12 points on the EAT-Lancet diet score, a binary index that features14 components expressed in grams. These results are similar to those found in our study. However, the two indices mentioned use a binary scoring system and do not allow a better distribution of the population's adherence to dietary recommendations [26]. The PHDI uses a gradual scoring system, in addition to being a caloric density index [12]. In this way, the PHDI allows a more adequate distinction between the degrees of adherence of individuals, favoring an interpersonal distribution and a more refined association with the outcomes evaluated.

In addition to the aforementioned studies, which evaluated the relationship between obesity indicators and EAT-Lancet-based indices, there was a population-based study, carried out by Seconda et al. [27], which assessed the relationship between the Sustainable Diet Index (SDI) and excess weight and obesity in French adults participating in the NutriNet-Santé study. The SDI is an index that features four sub-indices (nutritional, environmental, economic, and food practices), which are scored from 1 to 5 points, resulting in an overall SDI score of 4 to 20 points. As a result, the authors observed that lower SDI scores were associated with a higher risk of obesity and being overweight, when compared to individuals with higher SDI scores [27]. 

Despite the limited number of studies evaluating the relationship between the EAT-Lancet recommendations or adherence to sustainable diets and obesity, there are studies that relate diets considered sustainable, such as the Mediterranean diet [28,29,30] and the DASH diet [31], with a lower risk of being overweight or obese [32,33,34]. Furthermore, following a plant-based diet can be healthy and sustainable as it tends to create lower GHGE emissions [31,35], as well as being associated with a lower risk of obesity. A prospective study [36] that evaluated a Plant-Based Dietary Index (PDI) with obesity, suggested that the consumption of more plant-based foods and limiting animal-based diets might reduce the risk of obesity [36]. 

However, despite the promising results, we observed that most of the population evaluated only achieved half of the possible points on the PHDI (0 to 150 points, mean population score: 60.4 points), indicating that this population had a low adherence to the recommendations for a healthy and sustainable diet [12]. A possible explanation for this may be the higher consumption of red meat by the Brazilian population [37,38]. In addition, low scores were reported in the nuts and whole grains groups, indicating low consumption of these foods [12]. However, even with the population reaching only half of the possible points, those individuals who demonstrated a higher adherence to the PHDI were less likely to be overweight or obese, indicating that following a healthy and sustainable diet can be beneficial to human health, in addition to planetary health.

Our study featured some strengths, such as its use of a validated index that associated higher overall dietary quality and lower GHGE emissions. In addition, we used data from a well-established multicenter cohort study in Brazil, which followed strict data collection and processing protocols. However, some limitations may be noted as well. For instance, these results must be considered within the context of the study design. This was a cross-sectional analysis, which can evaluate association, but not causality. In addition, food consumption was assessed using an FFQ, an instrument that, despite being one of the most commonly used methods in epidemiological studies for evaluating the relationship between diet and health outcomes, still features some limitations, such as the finitude of its foods list and dietary misreporting bias. ELSA-Brasil is designed to assess risk factors and associations with cardiovascular diseases and diabetes, but it is not a population-based study, despite being a multicenter study from six different Brazilian cities across three major Brazilian regions. However, this characteristic allowed the inclusion of a population whose ethnic and social diversity was similar to that of heterogeneous populations, mainly of middle income, who live in large cities in Brazil. Thus, our external validity can be extended to urban centers with similar characteristics inside and outside Brazil.

In conclusion, we observed that higher PHDI scores were inversely associated with excess weight and obesity, as measured by both BMI and WC. These results demonstrate that the recommendations for a healthy and sustainable diet proposed by the EAT-Lancet have beneficial effects on human health, as they decrease the likelihood of being overweight or obese, which are risk factors for non-communicable chronic diseases, such as diabetes and cardiovascular disease. Furthermore, these results can offer important support for public policy planning and guidelines on the benefits of healthy and sustainable diets, with an emphasis on the proposal by the EAT-Lancet Commission.

## Figures and Tables

**Table 1:** Baseline characteristics of participants according to quintiles of the Planetary Health Diet Index. ELSA-Brasil, 2008–2010.

	Planetary Health Diet Index					
	1st	2th	3th	4th	5th	*p*-Value
n (mean)	2898 (44.7)	2913 (53.8)	2904 (59.9)	2889 (66.2)	2911 (76.8)	
Age group, n (%)						<0.001
Adults	2328 (80.3)	2345 (80.5)	2307 (79.4)	2280 (78.9)	2134 (73.3)	
Elderly	570 (19.7)	568 (19.5)	597 (20.6)	609 (21.8)	777 (26.7)	
Sex, n (%)						0.223
Men	1344 (46.4)	1332 (45.7)	1305 (44.9)	1337 (46.3)	1274 (43.8)	
Woman	1554 (53.6)	1581 (54.3)	1599 (55.1)	1552 (53.7	1637 (56.2)	
Self-reported race, n (%)						<0.001
White	1421 (49.0)	1452 (49.9)	1540 (53.0)	1545 (53.5)	1587 (54.5)	
Brown	902 (31.1)	864 (29.7)	806 (27.8)	787 (27.7)	739 (25.4)	
Black	477 (16.5)	500 (17.2)	463 (15.9)	454 (15.7)	464 (15.9)	
Other	98 (3.4)	97 (3.3)	95 (3.3)	103 (3.6)	121 (4.2)	
Per capita income, n (%)						<0.001
Low	1126 (38.9)	1123 (38.6)	1125 (38.7)	1067 (36.9)	924 (31.7)	
Medium	972 (33.5)	1027 (35.3)	956 (32.9)	992 (34.3)	1031 (35.4)	
High	800 (27.6)	763 (26.2)	823 (28.3)	830 (28.7)	956 (32.8)	
Smoking, n (%)						<0.001
Never	2442 (84.3)	2533 (87.0)	2521 (86.8)	2555 (88.4)	2577 (88.5)	
Current	456 (15.7)	380 (13.0)	383 (13.2)	334 (11.6)	334 (11.5)	
Excessive alcohol consumption, n (%)						0.002
No	2493 (86.0)	2505 (86.0)	2534 (86.9)	2485 (86.0)	2587 (88.9)	
Yes	405 (14.0)	408 (14.0)	380 (13.1)	404 (14.0)	324 (11.1)	
Physical activity, n (%)						<0.001
Low	2353 (81.2)	2302 (79.0)	2258 (77.8)	2200 (76.2)	2059 (70.7)	
Moderate	320 (11.0)	359 (12.3)	387 (13.3)	424 (14.7)	523 (18.0)	
Vigorous	225 (7.8)	252 (8.7)	259 (8.9)	265 (9.2)	329 (11.3)	
Diabetes type 2, n (%)						<0.001
No	2505 (86.4)	2467 (84.7)	2454 (84.5)	2359 (81.7)	2248 (77.2)	
Yes	393 (13.6)	446 (15.3)	450 (15.5)	530 (18.4)	663 (22.8)	
Hypertension, n (%)						0.009
No	1891 (65.3)	1906 (65.4)	1914 (65.9)	1839 (65.7)	1794 (61.6)	
Yes	1007 (34.8)	1007 (34.6)	990 (34.1)	1050 (36.3)	1117 (38.4)	
Dyslipidemia, n (%)						0.694
No	1235 (42.6)	1253 (43.0)	1198 (41.2)	1217 (42.1)	1219 (41.9)	
Yes	1663 (57.4)	1660 (57.0)	1706 (58.8)	1672 (57.9)	1692 (58.1)	
BMI (kg/m^2^), mean (sd)	27.1 (4.7)	27.0 (4.8)	26.9 (4.7)	27.2 (4.8)	26.8 (4.7)	<0.001
WC (cm), mean (sd)	91.7 (12.7)	91.3 (12.7)	90.7 (12.7)	91.4 (12.9)	90.6 (12.7)	<0.001
Energy intake (kcal), mean (sd)	1954.4 (755.6)	1971.7 (718.4)	1948.4 (702.1)	1936.7 (670.4)	1859.7 (607.7)	<0.001
BMI status, n (%)						0.002
Adequate	1013 (35.0)	1085 (37.3)	1097 (37.8)	1020 (35.3)	1152 (39.6)	
Overweight	1243 (42.9)	1143 (39.2)	1134 (39.1)	1190 (41.2)	1129 (38.8)	
Obesity	642 (22.2)	685 (23.5)	673 (23.2)	679 (23.5)	630 (21.6)	
Abdominal obesity, n (%)						0.050
Adequate	1036 (35.8)	1081 (37.1)	1111 (38.3)	1063 (36.8)	1097 (37.7)	
Increased abdominal obesity	803 (27.7)	730 (25.1)	749 (25.7)	756 (26.2)	805 (27.7)	
Substantially increased abdominal obesity	1058 (36.5)	1101 (37.8)	1044 (36.0)	1068 (37.0)	1008 (34.6)	

Quintiles: min–max—1st: 23.7–50.4; 2th: 50.5–56.9; 3th: 57.0–62.9; 4th: 63.0–69.9; 5th: 70.0–109.9.

**Table 2:** Multiple linear regression of the association between the Planetary Health Diet Index and obesity indicators. ELSA-Brasil, 2008–2010.

	Planetary Health Diet Index						
	Quintiles						Continuous (10-Point Increase in the Total Score)
	1st	2th	3th	4th	5th	*p* for Trend	
		β (95% CI)	β (95% CI)	β (95% CI)	β (95% CI)		β (95% CI)
BMI (kg/m^2^)							
Model age-adjusted ^£^	ref	−0.06 (−0.30:0.17)	−0.22 (−0.46:0.02)	0.04 (−0.20:0.28)	−0.42 (−0.66:−0.18)	0.007	−0.12 (−0.19:−0.06)
Model fully adjusted ^§^	ref	−0.11 (−0.34:0.12)	−0.25 (−0.48:−0.02)	−0.04 (−0.27:0.19)	−0.50 (−0.73:−0.27)	<0.001	−0.15 (−0.21:−0.08)
WC (cm)							
Model age-adjusted ^£^	ref	−0.40 (−1.04:0.24)	−1.13 (-1.77:−0.49)	−0.54 (−1.18:0.09)	−1.72 (−2.37:−1.09)	<0.001	−0.52 (−0.70:−0.35)
Model fully adjusted ^§^	ref	−0.44 (−1.02:0.14)	−1.10 (−1.68:−0.53)	−0.76 (−0.134:−0.18)	−1.70 (−2.28:−1.12)	<0.001	−0.52 (−0.68:−0.36)

Q1: 23.7–50.4; Q2: 50.5–56.9; Q3: 57.0–62.9; Q4: 63.0–69.9; Q5: 70.0–109.9. ^£^ Model adjusted for age. ^§^ Model adjusted for age, sex, self-reported race, income, smoking, excessive alcohol consumption, physical activity, diabetes, hypertension, dyslipidemia, energy intake, and dietary changes in the last six months.

**Table 3:** Multiple multinomial logistic regression between Planetary Health Diet Index and overweight and obesity. ELSA-Brasil, 2008–2010.

	Overweight *		Obesity *	
	OR	95% CI	OR	95% CI
Model age-adjusted ^£^				
PHDI quintiles				
1st quintile	ref		ref	
2th quintile	0.86	0.77:0.97	1.00	0.87:1.14
3th quintile	0.85	0.76:0.95	0.95	0.83:1.09
4th quintile	0.93	0.83:1.05	1.02	0.89:1.17
5th quintile	0.76	0.68:0.86	0.82	0.72:0.94
p for trend	<0.001		<0.05	
Continuous (10 points increase in the total score)	0.94	0.91:0.97	0.94	0.91:0.98
Model fully adjusted ^§^				
PHDI quintiles				
1st quintile	ref		ref	
2th quintile	0.85	0.76:0.96	0.97	0.84:1.13
3th quintile	0.84	0.75:0.95	0.95	0.82:1.10
4th quintile	0.92	0.82:1.04	0.97	0.84:1.13
5th quintile	0.76	0.67:0.85	0.76	0.65:0.88
p for trend	<0.001		<0.001	
Continuous (10 points increase in the total score)	0.93	0.90:0.97	0.92	0.88:0.96

OR: odds ratio. 95% CI: 95% confidence interval. * ref = BMI < 25 kg/m^2^. ^£^ Model adjusted for age. ^§^ Model adjusted for age, sex, self-reported race, income, smoking, intake excessive alcohol consumption, physical activity, diabetes, hypertension, dyslipidemia, energy intake, and dietary changes in the last six months.

**Table 4:** Multiple multinomial logistic regression between Planetary Health Diet Index and overweight and obesity. ELSA-Brasil, 2008–2010.

	Increased Abdominal Obesity *		Substantially Increased Abdominal Obesity *	
	OR	95% CI	OR	95% CI
Model age-adjusted ^£^				
PHDI quintiles				
1st quintile	ref		ref	
2th quintile	0.86	0.75:0.97	0.99	0.87:1.11
3th quintile	0.85	0.75:0.97	0.88	0.78:0.99
4th quintile	0.89	0.78:1.01	0.93	0.82:1.04
5th quintile	0.87	0.76:0.99	0.80	0.71:0.90
p for trend	0.095		<0.001	
Continuous (10 points increase in the total score)	0.96	0.93:0.99	0.93	0.90:0.97
Model fully adjusted ^§^				
PHDI quintiles				
1st quintile	ref		ref	
2th quintile	0.86	0.75:0.98	0.97	0.85:1.11
3th quintile	0.84	0.74:0.96	0.86	0.75:0.98
4th quintile	0.88	0.77:1.00	0.89	0.77:1.01
5th quintile	0.86	0.75:0.98	0.73	0.64:0.83
p for trend	0.066		<0.001	
Continuous (10 points increase in the total score)	0.95	0.92:0.99	0.91	0.87:0.94

OR: odds ratio. 95% CI: 95% confidence interval. * ref = WC < 80 cm for women and <90 cm for men. ^£^ Model adjusted for age. ^§^ Model adjusted for age, sex, self-reported race, income, smoking, intake excessive alcohol consumption, physical activity, diabetes, hypertension, dyslipidemia, energy intake, and dietary changes in the last six months.

## Supplementary Materials

The following Supplementary Materials are available online at https://www.mdpi.com/article/10.3390/nu13113691/s1. Table S1. Foods and ingredients included in the PHDI components; Table S2. Multiple linear regression of the association between the Planetary Health Diet Index and obesity indicators without under and overreporting of energy intake. ELSA-Brasil, 2008–2010; Table S3. Multiple multinomial logistic regression between Planetary Health Diet Index and excess weight and obesity without under- and over-reporting of energy intake. ELSA-Brasil, 2008–2010; Table S4. Multiple multinomial logistic regression between Planetary Health Diet Index and abdominal obesity without under- and over-reporting of energy intake. ELSA-Brasil, 2008–2010.
