# Supplementary material for: Adherence to the Planetary Health Diet Index and Obesity Indicators in the Brazilian Longitudinal Study of Adult Health (ELSA-Brasil)
Source: Nutrients. 2021 Oct 20;13(11):3691. doi: 10.3390/nu13113691 (PMC8625681; doi:10.3390/nu13113691)
Supplement: Supplementary file 1 [file nutrients-13-03691-s001.zip › nutrients-1408916-supplementary.pdf]

**Table S1.** Foods and ingredients included in the PHDI components.

| Components | Food items |
|---|---|
| Nuts and peanuts | Nuts, pistachios, almonds, peanuts, and coconut pulp and milk |
| Legumes¢ | Beans, pulses, lentils, chickpeas, peas, and soybeans. |
| Fruits | All fruits, including fruits used in juices, nectars and punces. |
| Vegetables | All vegetables |
| Whole cereals | Brown rice, whole breads, and oat flakes |
| Eggs | Chicken eggs and other poultry |
| Fish and seafood | Fish and seafood, such as mussels and shrimp |
| Tubers and potatoes | All types of potatoes and cassava as well as their derivatives flours |
| Dairy§ | Cow, goat, buffalo milk, yogurt, and cheese |
| Vegetable oilsº | Vegetable oils used in recipes, such as soybean oil, sunflower oil and olive oil. |
| DGV/total ratio≠ | Broccoli, arugula, cabbage, chicory, and watercress |
| ReV/total ratio≡ | Tomatoes, beetroots, carrots, and pumpkins |
| Red meat£ | Beef, lamb, pork, and processed meats |
| Chicken and substitutes | Chicken, poultry and excess substitutes (ie eggs, fish and seafood) |
| Animal fatsǂ | Butter and creams (e.g., sour cream and cheese cream) |
| Added sugars | All sweetener intake, including the sugars used as ingredients in processed or culinary products and the table sugar added to foods and beverages |

**Table 2.** Multiple linear regression of the association between the Planetary Health Diet Index and obesity indicators without under and overreporting of energy intake. ELSA-Brasil, 2008-2010.

| | Planetary Health Diet Index | | | | | | |
|---|---|---|---|---|---|---|---|
| | **Quintilesº** | | | | | | Continuous (10 points increase in the total score) |
| | **1st** | **2th** | 3th | 4th | 5th | p for trend | |
| | | β (95% CI) | β (95% CI) | β (95% CI) | β (95% CI) | | β (95% CI) |
| **BMI (kg/m²)** | | | | | | | |
| *Model age-adjusted£* | ref | -0.08 (-0.33:0.17) | -0.22 (-0.46:0.03) | 0.05 (-0.20:0.30) | -0.38 (-0.63:-0.13) | 0.026 | -0.11 (-0.18:-0.04) |
| *Model fully adjusted§* | ref | -0.13 (-0.36:0.10) | -0.25 (-0.48:-0.02) | -0.07 (-0.30:0.17) | -0.50 (-0.74:-0.27) | <0.001 | -0.14 (-0.21:-0.08) |
| **WC (cm)** | | | | | | | |
| *Model age-adjusted£* | ref | -0.36 (-1.01:0.30) | -1.08 (-1.73:-0.42) | -0.50 (-1.15:0.16) | -1.54 (-2.19:-0.88) | <0.001 | -0.48 (-0.66:-0.30) |
| *Model fully adjusted§* | ref | -0.44 (-1.02:0.14) | -1.04 (-1.62:-.046) | -0.81 (-1.39:-0.23) | -1.66 (-2.25:-1.08) | <0.001 | -0.48 (-0.66:-0.30) |

£Model adjusted for age. §Model adjusted for age, sex, self-reported race, income, smoking, alcohol intake, physical activity, diabetes, hypertension, dyslipidemia, energy intake and dietary changes in the last six months.

**Table S3.** Multiple multinomial logistic regression between Planetary Health Diet Index and overweight and obesity without under and overreporting of energy intake. ELSA-Brasil, 2008-2010.

| | **Overweight*** | | **Obesity*** | |
|---|---|---|---|---|
| | **OR** | 95% CI | OR | 95% CI |
| **Model age-adjusted£** | | | | |
| *PHDI quintiles* | | | | |
| 1st quintile | ref | | ref | |
| 2th quintile | 0.86 | 0.76:0.96 | 0.99 | 0.86:1.14 |
| 3th quintile | 0.83 | 0.74:0.94 | 0.97 | 0.84:1.11 |
| 4th quintile | 0.93 | 0.83:1.05 | 1.02 | 0.89:1.17 |
| 5th quintile | 0.76 | 0.68:0.86 | 0.83 | 0.72:0.96 |
| *p for trend* | <0.001 | | <0.05 | |
| *Continuous (10 points increase in the total score)* | 0.93 | 0.90:0.96 | 0.92 | 0.88:0.96 |
| **Model fully adjusted§** | | | | |
| *PHDI quintiles* | | | | |
| 1st quintile | ref | | ref | |
| 2th quintile | 0.85 | 0.76:0.96 | 0.96 | 0.83:1.11 |
| 3th quintile | 0.84 | 0.74:0.95 | 0.95 | 0.82:1.10 |
| 4th quintile | 0.92 | 0.80:1.03 | 0.96 | 0.83:1.12 |
| 5th quintile | 0.75 | 0.66:0.85 | 0.76 | 0.65:0.88 |
| *p for trend* | <0.001 | | <0.001 | |
| *Continuous (10 points increase in the total score)* | 0.93 | 0.90:0.96 | 0.92 | 0.88:0.96 |

OR: odds ratio. 95% CI: 95% confidence interval. *ref = WC<80cm for women and <90cm for men. £Model adjusted for age. §Model adjusted for age, sex, self-reported race, income, smoking, alcohol intake, physical activity, diabetes, hypertension, dyslipidemia, energy intake and dietary changes in the last six months.

**Table S4.** Multiple multinomial logistic regression between Planetary Health Diet Index and abdominal obesity without under and overreporting of energy intake. ELSA-Brasil, 2008-2010.

| | **Increased abdominal obesity*** | | **Substantially increased abdominal obesity*** | |
|---|---|---|---|---|
| | **OR** | 95% CI | OR | 95% CI |
| **Model age-adjusted£** | | | | |
| *PHDI quintiles* | | | | |
| 1st quintile | ref | | ref | |
| 2th quintile | 0.87 | 0.76:0.99 | 0.99 | 0.88:1.12 |
| 3th quintile | 0.87 | 0.76:0.99 | 0.91 | 0.80:1.03 |
| 4th quintile | 0.89 | 0.78:1.02 | 0.94 | 0.83:1.06 |
| 5th quintile | 0.90 | 0.79:1.03 | 0.82 | 0.72:0.93 |
| *p for trend* | 0.138 | | <0.001 | |
| *Continuous (10 points increase in the total score)* | 0.97 | 0.93:1.00 | 0.94 | 0.90:0.97 |
| **Model fully adjusted§** | | | | |
| *PHDI quintiles* | | | | |
| 1st quintile | ref | | ref | |
| 2th quintile | 0.86 | 0.75:0.99 | 0.97 | 0.85:1.11 |
| 3th quintile | 0.85 | 0.75:0.98 | 0.87 | 0.76:0.99 |
| 4th quintile | 0.88 | 0.77:1.00 | 0.88 | 0.77:1.01 |
| 5th quintile | 0.87 | 0.76:0.99 | 0.73 | 0.64:0.84 |
| *p for trend* | 0.098 | | <0.001 | |
| *Continuous (10 points increase in the total score)* | 0.95 | 0.92:0.99 | 0.91 | 0.88:0.94 |

OR: odds ratio. 95% CI: 95% confidence interval. *ref = WC<80cm for women and <90cm for men. £Model adjusted for age. §Model adjusted for age, sex, self-reported race, income, smoking, alcohol intake, physical activity, diabetes, hypertension, dyslipidemia, energy intake and dietary changes in the last six months.
